# The Influence of Accelerated Carbonation on Physical and Mechanical Properties of Hemp-Fibre-Reinforced Alkali-Activated Fly Ash and Fly Ash/Slag Mortars

**DOI:** 10.3390/polym14091799

**Published:** 2022-04-28

**Authors:** Ildiko Merta, Bojan Poletanovic, Jelena Dragas, Vedran Carevic, Ivan Ignjatovic, Miroslav Komljenovic

**Affiliations:** 1 Institute of Material Technology, Building Physics, and Building Ecology, Faculty of Civil Engineering, TU Wien, 1040 Vienna, Austria; bojan.poletanovic@tuwien.ac.at; 2 Faculty of Civil Engineering, University of Belgrade, 11000 Belgrade, Serbia; jelenad@imk.grf.bg.ac.rs (J.D.); vedran@imk.grf.bg.ac.rs (V.C.); ivani@imk.grf.bg.ac.rs (I.I.); 3 Institute for Multidisciplinary Research, University of Belgrade, 11030 Belgrade, Serbia; miroslav.komljenovic@imsi.rs

**Keywords:** accelerated carbonation, alkali-activated mortar, hemp fibres, natural fibres, fly ash, granulated blast furnace slag, physical properties, mechanical properties, energy absorption capacity

## Abstract

The physical and mechanical properties of hemp-fibre-reinforced alkali-activated (AA) mortars under accelerated carbonation were evaluated. Two matrices of different physical and chemical properties, i.e., a low Ca-containing and less dense one with fly ash (FA) and a high Ca-containing and denser one with FA and granulated blast furnace slag (GBFS), were reinforced with fibres (10 mm, 0.5 vol% and 1.0 vol%). Under accelerated carbonation, due to the pore refinement resulting from alkali and alkaline earth salt precipitation, AA hemp fibre mortars markedly (20%) decreased their water absorption. FA-based hemp mortars increased significantly their compressive and flexural strength (40% and 34%, respectively), whereas in the denser FA/GBFS matrix (due to the hindered CO_2_ penetration, i.e., lower chemical reaction between CO_2_ and pore solution and gel products), only a slight variation (±10%) occurred. Under accelerated carbonation, embrittlement of the fibre/matrix interface and of the whole composite occurred, accompanied by increased stiffness, decreased deformation capacity and loss of the energy absorption capacity under flexure. FA-based matrices exhibited more pronounced embrittlement than the denser FA/GBFS matrices. A combination of FA/GBFS-based mortar reinforced with 0.5 vol% fibre dosage ensured an optimal fibre/matrix interface and stress transfer, mitigating the embrittlement of the material under accelerated carbonation.

## 1. Introduction

Since 2020, the global coronavirus pandemic has impacted mobility and resulted in movement restrictions and work limitations across the globe. As the global economy slowed down during this period, great reductions in CO_2_ emissions were detected around the world [1,2]. Even though the focus of the main global targets was shifted toward ending the global pandemic, it became clearer than ever that human activities cause negative climate changes.

A responsible pathway for the mitigation of the serious effects of anthropogenic climate changes needs the introduction of actions in all spheres of life; thus, sustainability needs to be acknowledged as the most important aim in the 21st century. Radical changes are needed for achieving the aim of the Paris Climate Agreement, which is to “hold the increase in the global average temperature to well below 2 °C above pre-industrial levels and to pursue efforts to limit the temperature increase to 1.5 °C above pre-industrial levels” [3]. The construction industry is, without any doubt, one of the greatest CO_2_ emitters. It is not a surprise to stumble upon a podcast in The Guardian, titled “Concrete—the most destructive material on Earth” [4]. Concrete is the most widely used construction material today—roughly 33 billion tons of concrete are produced globally each year, or over 4.1 tons per person per year [5]. The production of concrete is responsible for 8% of global anthropogenic greenhouse gas emissions [6]. Cement production is a major source of carbon dioxide emissions—on average, 875 kg of CO_2_ per ton of clinker [6]. Approximately 60% of this amount is emitted from the calcination process of limestone, and the rest comes from the burning of fuels in the clinker kiln.

A significant reduction in the CO_2_ footprint from cement production is only possible with the reduction of the total cement mass used in concrete production. One of the most common approaches to reach this target is by partial or complete substitution of cement with supplementary cementitious materials (SCMs) with low embodied CO_2_ or by replacing the total cement amount with SCM in the production of alkali-activated (AA) binders and concrete. The most common materials used in the production of AA binders and concrete are by-products from other industries, such as fly ash (FA) from the combustion of coal in thermal power plants and granulated blast furnace slag (GBFS) from the production of iron in blast furnaces. Similarly to cement-based materials, AA materials show a significant brittle behaviour under tensile stresses. One of the ways to increase their energy absorption capacity is to reinforce them with short random fibres (such as steel, glass, carbon, synthetics, etc.) [7,8,9]. To foster sustainability in concrete construction, these traditional fibres should be replaced with more sustainable alternatives, such as, for example, natural fibres of hemp, flax, sisal, cotton, palm, raffia, etc. [10,11,12,13]. They are globally available, renewable, biodegradable, have low cost and their manufacturing processes are highly energy-efficient. Natural fibres have similar geometry and a comparable range of tensile strength and modulus of elasticity to some of the conventional synthetic fibres [14]. Natural fibres have been so far widely used in the automotive and textile industries; however, their employment as reinforcements in cementitious building materials is still under development. One of the major challenges for their successful application is still the assurance of their durability and long-term performance in the alkaline environment of the cementitious matrix [15,16]. When in contact with an alkaline matrix, natural fibres start to degrade. The two main mechanisms of fibre degradation are the alkaline attack on fibres and fibre mineralisation [15,16]. In the first mechanism, the alkaline pore water from the matrix dissolves the lignin and hemicellulose in fibres, which leads to the degradation of the fibres, whereas, during fibre mineralisation, the calcium hydroxide from the matrix destroys the fibre walls. Consequently, both mechanisms may lead to the composites’ compressive and flexural strength reduction and loss of their energy absorption capacity. However, the degradation of natural fibres within AA materials is still not known in regard to their degradation mechanism and rate, as well as the influence of carbonation on the natural fibre. In AA materials, carbonation is a chemically controlled reaction that occurs in two steps. During the first step, the carbonation of the pore solution leads to a decrease in the pH value and the eventual precipitation of Na-rich carbonates. This is followed by the decalcification of Ca-rich phases (mainly C-S-H, because Ca(OH)_2_ usually does not form in these systems) and by the carbonation of secondary reaction products present in the system [17]. Reaction products of AA materials (N-A-S-H, C-S-H, C-A-S-H or C(N)-A-S-H gels) depend on the employed precursor and alkaline activator type [17]. Thus, the carbonation of an AA material depends on its starting compounds’ composition [18].

The reaction of AA materials based on FA (ASTM Class F) with N-A-S-H gels and carbon dioxide (CO_2_) is mainly controlled by the carbonation of the pore solution, which leads to a pH value reduction and the precipitation of Na-rich carbonates. In the case of the systems based on GBFS (high calcium content), a partial decalcification of Ca-rich phases (C-A-S-H) has been reported, which can decrease the composite’s compressive strength [19]. A very limited number of available studies deal with the carbonation resistance of AA materials, and the results from the literature show opposing conclusions. Concretes based on FA [20] or GBFS [21] show similar or lower carbonation resistance compared to cement-based concrete. Some studies have shown that there may be an increase in the compressive strength of AA concrete due to carbonation [22]. These results are attributed to the refinement of the pore structure in AA materials, associated with the precipitation of carbonates forming as the carbonation reaction progresses [17]. Moreover, a minor strength degradation is observed, which intensifies with the increase in calcium content, i.e., by using high-calcium FA [20]. On the other hand, carbonation of the AA materials based on GBFS can lead to considerable structural strength degradation and an increase in porosity, particularly when the sodium silicate (water glass) is used for activation [23,24]. In the case of FA and GBFS blends, the carbonation resistance was reported to be similar to the carbonation of AA materials based on individual precursors [25]. Based on a systematic literature review, there is no research yet published dealing with the carbonation resistance of natural-fibre-reinforced AA materials.

The present work is a continuation of a previously published work [26] dealing with the physical and mechanical properties of hemp-fibre-reinforced AA fly ash and fly ash/slag mortars. The objective and main novelty of the present work is the experimental evaluation of the influence of accelerated carbonation on these mortars. The same two types of mortars (differing in their chemical composition and physical properties, such as density, compactness and porosity) were used. The first mortar was made with FA solely, whereas the second was made with the combination of FA and GBFS. The matrices were made with the same type of alkali activator, cured under the same conditions (temperature and humidity) and reinforced with short hemp fibres of 10 mm in length, with two fibre dosages (0.5 vol%, 1.0 vol%) and exposed to accelerated carbonation. A thorough literature review shows that the results presented here are most likely the first ones dealing with the accelerated carbonation of natural-fibre-reinforced AA mortars and this is one of the few works in the area of natural-fibre-reinforced AA mortars [26,27,28,29,30].

## 2. Experimental Method

This research is a continuation of previously published results [26] where the physical and mechanical properties of hemp-fibre-reinforced AA mortars made with two different matrices and two fibre amounts at the age of 28 days were evaluated.

### 2.1. Materials

The AA mortars tested in this study were made using locally available waste materials, i.e., SCMs: two different samples of FA and one sample of GBFS. Both FAs (F1 and F2) were sampled from the same coal-fired power plant, “Nikola Tesla B”, in Obrenovac, Serbia, and used without further treatment. GBFS was obtained from pig iron production at the facility “Zelezara Smederevo” (Serbia) and additionally ground to its specific surface area of 400 m^2^/kg (according to the Blaine test). Both FA samples correspond to the ASTM-C618 Class F [31], as they have similar chemical composition but different particle size distribution [26]. The particle size distribution of FA and GBFS samples, density and the main part of their chemical composition are shown in Table 1.

**Figure 1:**
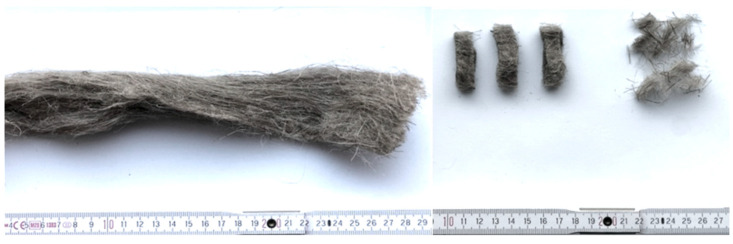
Hemp fibres.

The AA mortars were prepared with a sodium silicate solution that had a modulus of *n* = SiO_2_/Na_2_O = 1.91 and a density of 1514 kg/m^3^. The samples were prepared with river sand (0–4 mm) of a water absorption of 0.385% and a dry density of 2670 kg/m^3^. The mortars were reinforced with primary industrial bast hemp fibres (*Cannabis sativa* L.) cultivated and processed (through the retting process) in Hungary. The fibre diameter was 8–60 μm, the density 1500 kg/m^3^, while the tensile strength ranged between 270 and 900 MPa [11], and the water absorption was 2.5 times the fibres’ weight. The fibre bundles were first combed and then cut with a scissor to a length of 10 mm (Figure 1).

### 2.2. Mortar Mix Design

Two different mortar matrices (FA1 and FA2S) of different compressive strength (governed by different chemical compositions and densities) were prepared and tested by using different mortar compositions and raw materials. The used mixtures were developed and optimised on different AA pastes in the framework of preliminary experiments in the previous research [26] based on the optimal hardening time, workability and compressive strength. 

The first group of mortar matrices (FA1) was made only from fly ash type F1, and the second one (FA2S) with the combination of fly ash type F2 and GBFS. The FA2S matrix was based on the markedly different chemical composition of the solid precursors, which contained smaller and more reactive particles, and thus this matrix was more compact, with a higher density and a lower porosity than the FA1 matrix. In both mortar matrices, two different hemp fibre dosages were used—0.5 vol% and 1.0 vol%—resulting in overall six different mortar matrix mixtures. In all matrices, the same amounts of sand (1350 g) and sodium silicate solution were used (306 g). The amount of water in the mortars was determined based on the similar workability and the additional water needed for the fibres’ uptake. The mortar mix design is shown in Table 2. 

Both mortar mixtures were prepared by mixing sodium silicate solution, water and fly ash F1 (for FA1) or fly ash F2 and GBFS (for FA2S). They were mixed for 60 s at low speed (40 ± 5 revolutions/min); then, within the next 30 s (rest period), sand, dry hemp fibres and the additional water (for fibres’ water uptake) were added, and the mixtures mixed for an additional 90 s at a medium speed (285 ± 5 revolutions/min) in order to achieve a homogeneous dispersion of the fibres within the matrix. Afterwards, mortar prism specimens of dimensions 40 × 40 × 160 mm^3^ were cast in moulds, vibrated and closed in plastic bags. Following a resting time of approximately one hour under controlled laboratory conditions (20 °C temperature, 50% relative humidity), the specimens were placed in a heating chamber at 80 °C for 24 h. The specimens were then kept in plastic bags and stored under laboratory conditions (20 °C temperature) until the age of 90 days, when the accelerating carbonation started.

### 2.3. Accelerated Carbonation

Natural carbonation is a very slow process (measured in years). Thus, in order to experimentally evaluate such influence in a reasonable time frame, and by assuming that the effects of accelerated carbonation are comparable to the effects of long-term natural carbonation, in this experimental campaign, accelerated carbonation was applied. The acceleration of the carbonation process was achieved by increasing the CO_2_ concentration. At the age of 90 days, the mortar prism samples were placed in a carbonation chamber. The accelerated carbonation was performed for 56 days at a CO_2_ concentration of 1%, relative humidity of 60 ± 10% and a temperature of 21 ± 2 °C according to the European standard for mortar samples EN 13295 [32].

### 2.4. Experiments

All measurements of the physical and mechanical properties of the specimens were conducted both prior to and after the accelerated carbonation. The density of the specimens was measured on four mortar prisms (40 × 40 × 160 mm^3^) and was calculated as follows:

D [kg/m^3^] = M/V (1)

where M is the mass and V is the volume of the specimens.

The three-point bending test (3PBT) was conducted according to the EN 1015-11 standard [33], on four specimens (of dimensions 40 × 40 × 160 mm^3^), whereas the compressive strength test was performed on the remaining half of the specimens after the 3PBT (of dimensions 40 × 40 × 40 mm^3^). The tests were carried out on a mechanical testing machine, the Zwick/Roell Z250, with a load capacity of 200 kN and a rigidity of 8 × 10^−3^ mm/kN, at room temperature of 21 °C and relative humidity of 50%. The 3PBT was performed under a controlled displacement of 400 μm/min (closed-loop test), whereas the compressive test was performed with the loading rate of 0.5 MPa/s. Under 3PBT, the force-displacement curves of the specimens were recorded. The specific energy absorption capacity of the specimens was calculated from the 3PBT, as the area under the force-displacement curve, which was divided by the specimens’ cross-section. The area was calculated by integration of the force-displacement curves with an upper limit of the displacement corresponding to the maximal force drop of 98%. In addition to this, the percentage of the corresponding value of energy absorption capacity in pre- and post-peak parts of the curve (before and after the peak) was calculated and compared.

The total water absorption test was conducted on the four halves of the mortar prism specimens of dimensions (40 × 40 × 80 mm^3^-obtained from the fractured specimens after the three-point bending test). The specimens were placed in a water bath, supported by a plastic holder on the bottom side to provide complete contact with water. After two days, when a water absorption equilibrium within the specimens was obtained, the water absorption was calculated as follows:

Mt [%] = (Wt − Wo) × 100/Wo (2)

where Wt is the mass of water-saturated specimens and Wo is the mass of dry specimens.

## 3. Results and Discussion

The physical and mechanical properties of the mortars before the accelerated carbonation have been analysed in detail and commented on in previous research [26]; however, for a reasonable comparison here, a short insight is also given. In the discussion of the force-midspan deflection diagrams and the energy absorption capacity of the mortars, however, more details in the previous results [26] were inevitable. In the present research, the “transition point (TP)” and the “failure point (FP)” terminologies were introduced and the results of [26] were discussed and compared with the present results from this perspective also.

**Figure 2:**
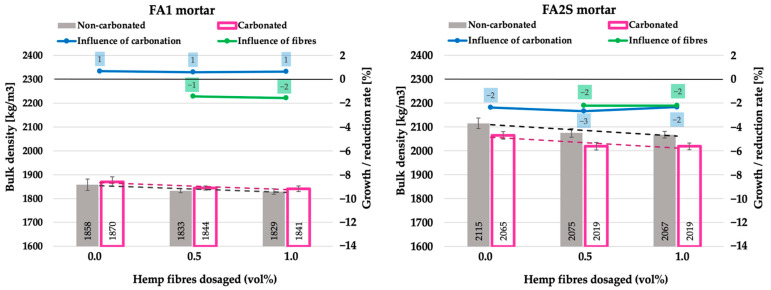
Density of fly-ash-based (FA1) and fly-ash-and-slag-based (FA2S) mortars prior to [26] and after accelerated carbonation.

**Figure 3:**
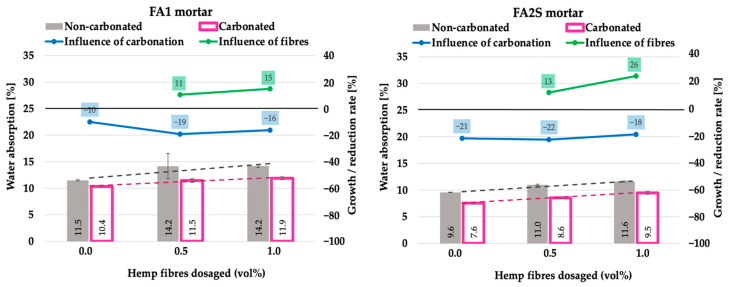
Total water absorption of fly-ash-based (FA1) and fly-ash-and-slag-based (FA2S) mortars prior to [26] and after accelerated carbonation.

**Figure 4:**
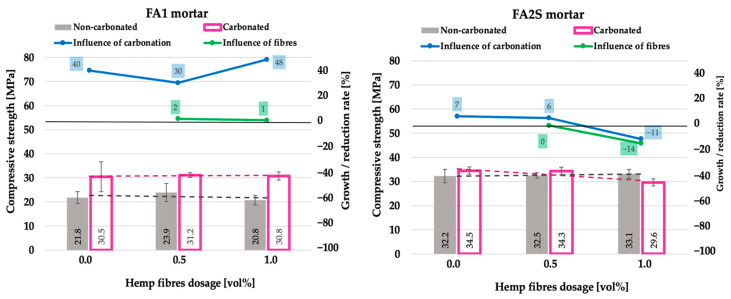
Compressive strength of fly-ash-based (FA1) and fly-ash-and-slag-based (FA2S) mortars prior to [26] and after accelerated carbonation.

The influence of accelerated carbonation on particular mortars is illustrated in the graphs (blue line in Figure 2, Figure 3, Figure 4, 6 and 7) by means of the growth/reduction rate [%], which represents the improvement or degradation (reduction) of a particular mechanical property under the accelerated carbonation. It is calculated as the quotient between the value after and before accelerated carbonation. In the case of density, compressive strength, flexural strength and energy absorption capacity, positive values indicate an improvement in the property, whereas negative values indicate degradation. Solely in the case of water absorption, the opposite applies. The influence of hemp fibre dosage in carbonated matrices is illustrated in the graphs (green line in Figure 2, Figure 3, Figure 4, 6 and 7) as the quotient between the value with and without fibres after accelerated carbonation.

Generally, in both mortars (FA1 and FA2S), the reinforcement with hemp fibres decreases slightly the mortar’s density [26]. This is due to the increased porosity caused by entrained air within the matrix during the mixing of fibres. Under accelerated carbonation, both hemp-fibre-reinforced mortars exhibited only a slight change in density (within the 5% margin) (Figure 2). 

The FA1 matrix showed negligible densification (up to 1% increase) whereas the FA2S matrix had a slight density decrease (up to 3%). The increase in the fibre’s volumetric dosage itself within the matrix has no influence on the density.

The composite’s water absorption capacity is directly correlated to its porosity. The majority of the total pore volume of the material is occupied by open pores, i.e., macropores and large capillary pores that are larger than approximately 1 μm. Since water is able to ingress only in these open pores, capturing this range of pores can give a sufficiently accurate estimation of the composite’s total porosity [26].

The long-term durability of the natural-fibre-reinforced cement-based and alkali-activated composites is impacted by humidity [34]. Water ingress into the matrix leads to fibre swallowing, which leads to the matrix cracking and fibre-matrix debonding [35]. Thus, in order to improve the composite’s long-term durability, it is important for the host matrix to have high density, low porosity and low water absorption.

In both matrices, the addition of hemp fibres resulted in an increase in the total water absorption of the specimens [26] (Figure 3).

This is, on one hand, due to the overall increased porosity of the matrix as a result of entrained air during the mixing of the fibres into it and, on the other hand, due to the fibres’ hydrophilic nature (relatively high water absorption capacity of hemp fibres, i.e., 2.5 times their weight) [26]. In non-carbonated mortars, slightly higher water absorption (on average 23%) was observed in the fly-ash-based FA1 matrix compared to the denser FA2S matrix (on average 18%).

Under accelerated carbonation in both matrices, a decrease in water absorption capacity was observed, which is favourable in terms of the composite’s long-term durability. The decrease in water absorption under accelerated carbonation results from the pore refinement of the matrix [36] as a consequence of different alkali and alkaline earth salts’ precipitation, such as sodium and calcium carbonate, which were formed during the carbonation reaction process. The precipitated reaction products refine the pores [37] and consequently hinder the water penetration. However, in the case of the denser matrix (FA2S), the decrease in water absorption is slightly more pronounced (on average 20%) than in the FA1 matrix (on average 17%). This is due to its higher initial material compactness and density, as well as lower porosity.

It is, however, interesting that when hemp-fibre-reinforced fly ash-based (FA1) matrices are exposed to accelerated carbonation, a more distinctive decrease in water absorption occurs than in the case of non-reinforced specimens, i.e., 19% (for 0.5 vol% hemp fibre dosages) and 16% (for 1.0 vol% fibres dosage) compared to 10% (for non-reinforced specimens). Thus, the reinforcement of the matrix with hemp fibres seems to improve the long-term durability of the composite under the action of accelerated carbonation. The reason for this could be that the hemp-fibre-reinforced mortars have higher air content (due to their higher porosity, indicated with higher water absorption capacity; Figure 3) that could be filled in with alkali and alkaline earth salts’ precipitation formed during the carbonation process. In the case of the denser matrix (FA2S) with initially much lower water absorption, however, hemp-fibre-reinforced and non-reinforced mortars exhibited the same water absorption decrease rate (of around 20%). In both carbonated mortars, the increase in the hemp fibre dosage itself (from 0.5 vol% to 1.0 vol%) resulted in an increase in water absorption under accelerated carbonation. In the denser FA2S matrix, a more pronounced increase in water absorption was observed than in the FA1 matrix.

In cement-based matrices reinforced with coconut fibres (1–3 vol% of fibre dosage), Bui et al. [38] report a similar trend under accelerated carbonation (12 weeks of exposure to 4% CO_2_ under 20 °C and 65% RH). The increased porosity as a result of entrained air induced through the mixing of coconut fibres in the matrix improved the carbonation rate of the mortars, densified the matrix and consequently decreased its porosity.

In both matrices (FA1 and FA2S), the addition of hemp fibre reinforcement did not have any marked influence on the compressive strength of the matrix [26] (Figure 4).

However, after accelerated carbonation, a notably different behaviour was observed between the two matrices. In FA1, a significant compressive strength improvement (on average 40%) occurred, whereas in FA2S, only a slight variation (up to ±10%) was observed. The reason for the pronounced strength improvement in the FA1 matrix is believed to be the more pronounced precipitation of alkali and alkaline earth salts from the pore solution and the refinement of the pore structure after accelerated carbonation [17,25]. The reaction between N-A-S-H gels (mainly formed in the fly-ash-based AA materials) and CO_2_ is mainly controlled by the carbonation of the pore solution, which leads to the precipitation of alkali and alkaline earth salts. The difference in the strength gain between FA1 and FA2S mortars after accelerated carbonation was also related to their different initial chemical and physical properties. FA2S mortars have a more compact matrix (higher density, lower porosity) [26], which hinders the penetration of the gaseous CO_2_ and consequently results in a lower chemical reaction between CO_2_ and the matrix’s pore solution and matrix’s gels. This was apparent from the slight density decrease of the FA2S matrix after accelerated carbonation (Figure 2), which consequently resulted in a lower compressive strength gain.

On the other hand, in matrices based on GBFS, a partial decalcification of Ca-rich phases (C-A-S-H) due to the carbonation process is reported in the literature [19], which can also result in a decrease in compressive strength. The FA2S matrix is based on two different solid precursors; thus, this might result in a relatively low compressive strength improvement (or even loss at higher fibre dosages) under accelerated carbonation. Under accelerated carbonation, the increase in hemp fibre dosage in the FA1 matrix did not have any impact on the compressive strength, whereas in the FA2S matrix, with increasing fibre dosage, the compressive strength decreased by 14%.

The main reason for such behaviour of the FA2S matrix most probably lies in the higher sensitivity of high calcium-containing systems to carbonation, i.e., the higher calcium content present in the FA2S matrix (combined with higher hemp fibre and water content) during the accelerated carbonation led to more pronounced matrix destruction when compared to a lower calcium-containing matrix such as FA1. Since the compressive strength is predominantly governed by the strength of the matrix itself (resulting from its density and porosity) and much less by the effect of the fibre, the increase in fibre dosage itself caused the more pronounced degradation in the compressive strength in the FA2S matrix than in the FA1.

Concerning the compressive strength of plain (non-reinforced) AA composites under accelerated carbonation, so far, contradictory results, i.e., both increases [22] and decreases [39,40], are reported in the literature. Deja [22] reports an increase in compressive strength under carbonation, whereas Bernal et al. [39] and Li et al. [40] report up to a 27% decrease in alkali-activated slags’ compressive strength under accelerated carbonation.

In the literature, so far, solely results of the compressive strength of either synthetic-fibre-reinforced AA mortars [41] or natural-fibre-reinforced cement-based matrices [38] under accelerated carbonation have been published. Generally, in both cementitious and AA fibre-reinforced composites, the addition of fibres increases the air content in the matrix, allowing easier CO_2_ penetration and consequently the precipitation of carbonation reaction products, which fill the pores and densify the matrix, resulting in an increase in compressive strength. In coconut-fibre-reinforced cement-based matrices (1–3 vol% of fibre dosage), Bui et al. reported up to a 32% increase in compression strength under accelerated carbonation (12 weeks of exposure to 4% CO_2_ under 20 °C and 65% RH), whereas in polyvinyl alcohol fibres (with up to 2.5 vol% fibre dosage) reinforced FA-based geopolymers (with up to 20% substitution by GBFS, metakaolin and silica fume), Li et al. [41] report even up to a 50% increase in compressive strength (56 days of exposure to 20 ± 3% CO_2_ under 20 ± 5 °C and 70 ± 5% RH).

**Figure 5:**
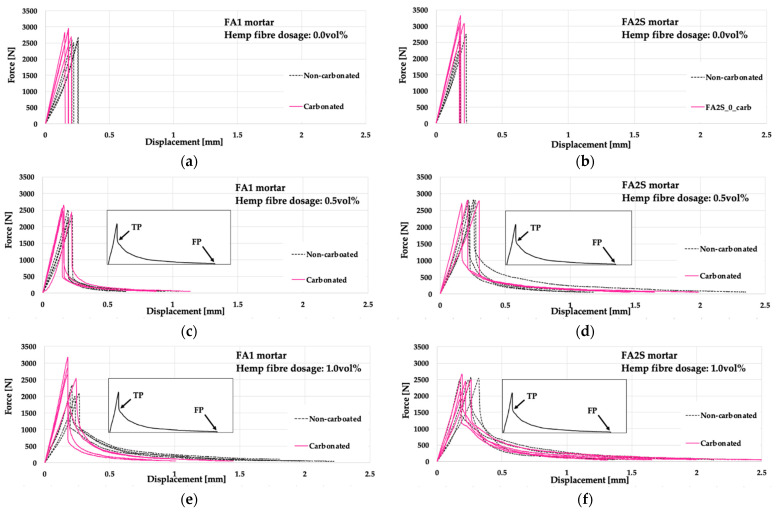
Force-midspan deflection curves of fly-ash-based (FA1) and fly-ash-and-slag-based (FA2S) mortars prior to [26] and after accelerated carbonation.

The force-midspan deflection diagrams of the mortars under 3PBT are given in Figure 5.

The specimens exhibited a typical quasi-brittle fracture behaviour by the initiation of one single discrete crack by reaching the peak load and a strain-softening behaviour in the post-peak region of the curve [26]. The peak of the curve represents the flexural strength of the composite. Generally, in fibre-reinforced composites, the fibres are activated in carrying stresses (by bridging the crack) first when the matrix is cracked [42], and they have no significant influence on the composite’s flexural strength.

**Figure 6:**
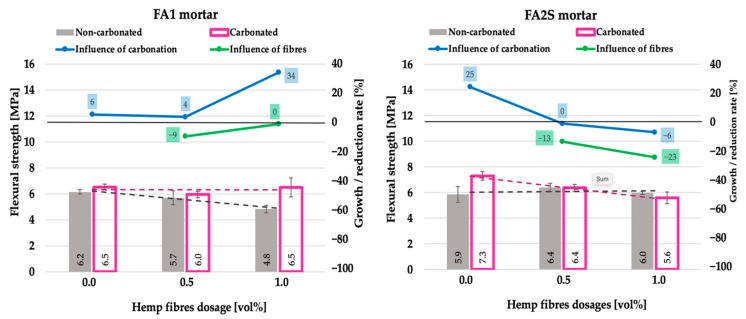
Flexural strength of fly-ash-based (FA1) and fly-ash-and-slag-based (FA2S) mortars prior to [26] and after accelerated carbonation.

Similar to the case of the compressive strength, the addition of hemp fibre reinforcement had no marked influence on the flexural strength [26] (Figure 6). 

In FA1 specimens, a slight decrease was observed, whereas in FA2S specimens, no change occurred. Under accelerated carbonation, the flexural strengths of all mortars followed a very similar trend to their compressive strengths. Again, in the FA1 matrix, a distinctive flexural strength improvement (up to 34%) occurred, whereas in the FA2S matrix, no change (or a slight decrease) was observed. The reason for this is the same mechanism as described for the compressive strength, resulting from the differences in the matrix’s chemical composition, density and porosity, which, in turn, govern the ease of the penetration of CO_2_.

Under accelerated carbonation, the increase in the hemp fibre dosage in the FA1 matrix had almost no impact on the flexural strength, whereas in the FA2S matrix, with increasing fibre dosage, the flexural strength decreased by up to 23%. This trend was once again following the same trend as the matrix’s compressive strength under accelerated carbonation.

From the force-midspan deflection diagrams of non-reinforced matrices (Figure 5a,b), it is apparent that after reaching a maximum force, a sharp drop in the force in the post-peak region occurred, indicating the negligible deformation and energy absorption capacity of the composite. In hemp fibre matrices (Figure 5c–f), however, a distinctive effect of the fibres on the post-peak behaviour is evident [26]. Following a sharp drop in the force in the post-peak region, a plateau of the curve develops, with a gradual force decrease under a significant increase in displacement (mid-span deflection). At the transition point (TP) (between the sharp vertical part of the curve and the plateau), the fibres in the cracked matrix start to be activated by providing stress transfer through bridging the cracks. Starting from the TP, the fibres are pulled out from the matrix channel, and through the fibre/matrix friction, the energy dissipation mechanism is activated (quantified by the area under the force-midspan deflection curve). The failure of the specimens is reached at the end of the plateau, i.e., at the failure point (FP), under significant displacement values.

**Figure 7:**
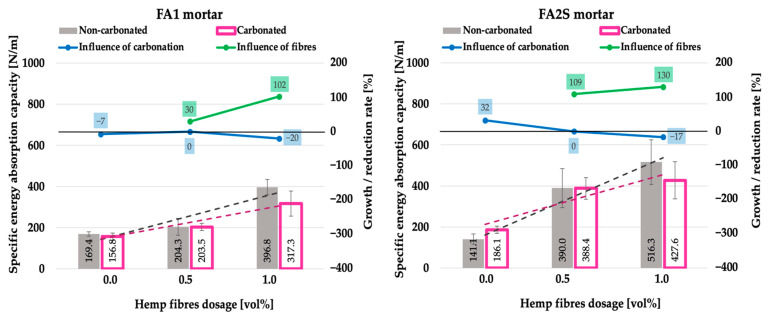
Specific energy absorption capacity of fly-ash-based (FA1) and fly-ash-and-slag-based (FA2S) mortars prior to [26] and after accelerated carbonation.

Concerning the energy absorption capacity of the composite, a marked improvement (an increase in the area under the force-midspan deflection curve) with the addition of hemp fibres was observed [26] (Figure 7). The fibres effectively prolong the crack propagation within the matrix and provide stress transfer through bridging the cracks. With increasing fibre dosage (1.0 vol%), the TP was higher positioned on the *y*-axis (Figure 5e,f) compared to the lower fibre dosage (0.5 vol%) (Figure 5c,d), which is the result of the fibre’s higher reinforcing ratio (more fibres per cross-section). Thus, with higher fibre dosages, more fibres were active in stress transfer, resulting in higher failure resistance and energy dissipation (Figure 7).

However, in the case of the denser FA2S matrix, the TPs were generally more highly located on the *y*-axis (Figure 5d,f) than by the corresponding FA1 matrix (for the same fibre dosage) (Figure 5c,e), indicating an earlier activation of the fibres in stress transfer. It resulted in a higher-lying plateau, which in turn results in a higher energy absorption capacity (area under the curve) of the FA2S matrix. It indicated that in the case of a denser matrix, a more optimal fibre/matrix interface and stress transfer occurred [26]. The increase in the fibre dosage itself (from 0.5 vol% to 1.0 vol%) improved, however, in a more pronounced manner the energy absorption capacity of the denser FA2S matrix than that of the FA1 matrix (higher inclination of the black dashed curve) (Figure 7). This again indicated a more optimal fibre/matrix interface in the denser matrix [26].

In all specimens, after accelerated carbonation, a steepening of the elastic part of the force-midspan deflection curve occurred (Figure 5). The composite became stiffer with lower elastic strain (lower deformation capacity), i.e., with a higher modulus of elasticity (steeper slope of the curve). Thus, as a consequence of the precipitation of alkali and alkaline earth salts from the pore solution under accelerated carbonation, embrittlement of the matrix occurs.

However, generally, in the FA1 matrix (Figure 5a), the effect of the embrittlement was more pronounced than in the denser FA2S matrix (Figure 5b). In the FA2S matrix, the displacement corresponding to the maximal force after accelerated carbonation remained almost the same but the slope of the curves’ elastic part slightly inclined, resulting at the end in a 32% increase in energy absorption capacity (Figure 7). In the FA1 matrix, on the contrary, the energy absorption capacity after accelerated carbonation decreased by 7% (Figure 7). This is because, in the FA2S matrix, a combination of embrittlement (alkali and alkaline earth salt precipitation due to the presence of FA and GBFS) and of a partial decalcification (of the Ca-rich phase due to the presence of GBFS) occurred. These two mechanisms oppositely influenced the embrittlement of the matrix and they either could balance each other out, or one of the mechanisms could counteract the other one. This could finally result in no change or even a decrease in the initial brittleness. Additionally, in the initially more compact and denser FA2S matrix, the diffusion of gaseous CO_2_ is much more hindered than in the FA1 matrix, limiting thus the accelerated carbonation rate within the matrix.

The difference in the embrittlement (sensitivity to embrittlement) in the FA1 and FA2S matrices is apparent in the case of reinforcement with hemp fibre also. Hemp fibre FA1 matrices exhibited more pronounced embrittlement than hemp fibre FA2S matrices. From the force-displacement curves of the FA2S matrix (Figure 5d,f), it is apparent that upon reaching the peak of the curve, a lower force drop occurred than by the FA1 matrix (Figure 5c,e). The fibre pull-out in the post-peak region started earlier (at 69% and 40% force drop for 0.5 vol% and 1.0 vol% of fibres, respectively) than in the FA1 matrix (at 70% and 46% force drop for 0.5 vol% and 1.0 vol% of fibres, respectively)—indicating an earlier activation of the fibres in stress transfer. This resulted consequently in a higher-lying plateau of the curves and finally in a higher energy absorption capacity in FA2S compared to FA1 (Figure 7). Thus, in carbonated specimens, again, in the case of a denser matrix, a more optimal fibre/matrix interface and stress transfer occurred.

Generally, under accelerated carbonation in all fibre-reinforced specimens, the plateau of the curves between TP and FP lay lower than by non-carbonated specimens, indicating lower effectiveness of the fibres in stress transfer, which in turn results in lower energy dissipation. Due to the embrittlement of the fibre/matrix interface as well after accelerated carbonation, a stronger fibre/matrix bond develops, which in turn results in more breakage of the fibres instead of being pulled out. Thus, after accelerated carbonation, the fibres lose their effectiveness in stress transfer.

Under accelerated carbonation, as a result of the embrittlement, generally, the FP occurs earlier than in non-carbonated specimens, indicating a lower deformation capacity of the composite as well. Under accelerated carbonation, the increase in hemp fibre dosage in the matrix (from 0.5 vol% to 1.0 vol%) has a negative effect on the energy absorption capacity of the composite. Namely, specimens reinforced with 0.5% hemp fibre reinforcement after accelerated carbonation retained completely their energy absorption capacity (0% loss, Figure 7), whereas specimens reinforced with 1.0 vol% hemp fibre reinforcement lost 17% and 20% (for FA2S and FA1 respectively) of their energy absorption capacity under accelerated carbonation. In the case of a higher fibre dosage (higher reinforcement ratio), more fibres were present in the cross-section, which in turn resulted in more contact points with the matrix. The embrittlement of the matrix under accelerated carbonation, however, resulted in a stronger fibre/matrix bond (even more contact points), which hindered the optimal development of the fibres’ pull-out mechanism and resulted in more fibres being broken instead of being pulled out.

**Figure 8:**
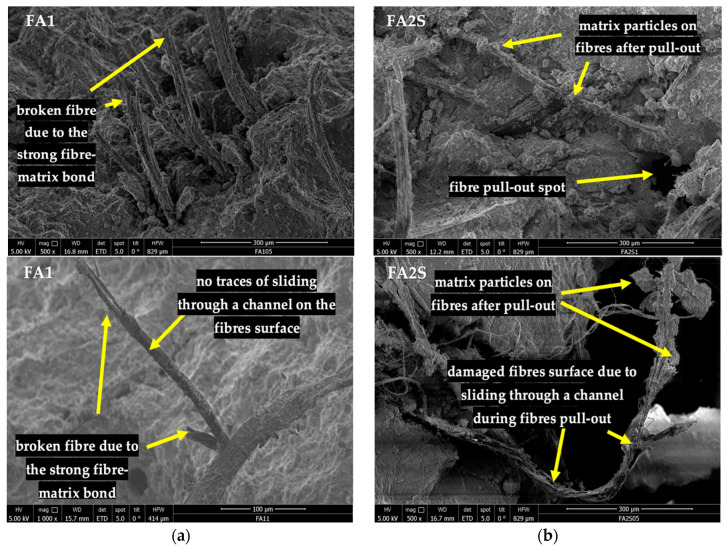
SEM images of fracture surfaces of fibre-reinforced AA mortars after accelerated carbonation for: (**a**) fly-ash-based AA mortar (FA1); (**b**) fly-ash-and-GBFS-based AA mortar (FA2S).

However, in the denser FA2S matrix, the degradation in the energy absorption capacity under accelerated carbonation was slightly lower, indicating again a more optimal fibre/matrix interface and stress transfer compared to the FA1 matrix. This is believed to be the result of the Ca-rich phases’ decalcification, which resulted in a loosening of the fibre/matrix bond (fewer contact points) and a beneficial effect on the fibres’ pull-out mechanism. This is apparent in the SEM pictures of the fracture surfaces (after 3PBT) of carbonated specimens, wherein the FA2S matrix (Figure 8b) fibre pull-out spots and matrix particles on the surfaces of the fibres could be noticed. This is a consequence of the fibres’ sliding through the matrix channel during the pull-out, which in turn results in higher energy absorption capacity. In the FA1 matrix (Figure 8a), on the contrary, no traces of matrix particles were observed and more broken fibres were present in the cross-section, which is a consequence of a stronger fibre/matrix bond.

Under accelerated carbonation, the addition of hemp fibres in the FA1 matrix increased the energy absorption capacity of the mortar by 30% and 102% for 0.5 vol% and 1.0 vol% fibre dosages, respectively. In the FA2S matrix, the increase was even higher. After 0.5 vol% and 1.0 vol% fibre dosages, the mortar’s energy absorption capacity increased by 109% and 130%, respectively. Based on these results, hemp fibre AA mortars with a denser matrix and lower fibre dosage (0.5 vol%) performed more optimally under accelerated carbonation in terms of the composite’s energy absorption capacity. To the best of the authors’ knowledge, no results of AA natural-fibre-reinforced mortar (or concretes) under accelerated carbonation have been published dealing with the behaviour of the composite under flexural load.

## 4. Conclusions

In this research, the influence of accelerated carbonation on the physical and mechanical properties of hemp-fibre-reinforced AA mortars was experimentally evaluated on two matrices in terms of different chemical and physical properties (FA-based matrix with lower calcium content and density and FA/GBFS-based with higher calcium content and density). Based on the obtained results, the following could be concluded:

- In both matrices, the accelerated carbonation had no significant influence (up to ±3%) on the density of hemp fibre AA mortars. In FA-based matrices, a slight densification occurred, whereas in FA/GBFS matrices, a slight decrease in density occurred.
- As a consequence of the pore refinement, the water absorption of both hemp fibre AA matrices decreased by up to 20% under accelerated carbonation. This is a favourable effect in terms of the composite’s long-term durability.
- Under accelerated carbonation in the FA-based hemp fibre matrix, a significant compressive and flexural strength increase (up to 40% and 34%, respectively) occurred, whereas in the denser (FA/GBFS) matrix, a much lower variation (up to ±10%) was observed. In the denser matrix, the penetration of the gaseous CO_2_ was hindered, which consequently resulted in a lower chemical reaction between CO_2_ and the pore solution and gel products of the matrix and in a lower strength improvement.
- As a consequence of the precipitation of alkali and alkaline earth salts from the pore solution under accelerated carbonation, embrittlement of the matrices accompanied by increased stiffness (higher modulus of the elasticity) and decreased deformation capacity (lower elastic strain) occurred. FA-based matrices exhibited more pronounced embrittlement than the denser FA/GBFS matrices.
- As a result of the embrittlement of the fibre/matrix interface under accelerated carbonation, the energy absorption capacity of hemp fibre specimens under flexure decreased. Due to the stronger fibre/matrix bond, the fibres lost their effectiveness in stress transfer, which led to more broken than pulled-out fibres. FA-based matrices exhibited a more pronounced fibre/matrix degradation. The increase in fibre dosage (from 0.5 vol% to 1.0 vol%) had a distinctly negative effect, resulting in an up to 20% decrease in the energy absorption capacity of the composites.

Based on these findings, hemp-fibre-reinforced AA mortars could represent a potential sustainable material in replacing traditional cement-based mortars in some applications. However, the main challenge in the application of bio-based building materials, in general, is their long-term durability, resulting from the potential degradation of plant-based materials in the alkaline environment [34,43,44]. This aspect should be carefully considered in the development and design of the composite, with a specific focus on the mitigation of the fibre/matrix interface degradation.

## Figures and Tables

**Table 1:** The particle size distribution, density and chemical composition of FA and GBFS.

Supplementary Cementitious Material Type	Average Mean Particle Size [μm]	Density [kg/m^3^]	Al_2_O_3_ [%]	SiO_2_ [%]	Fe_2_O_3_ [%]	CaO [%]
F1	16.78	1960	18.47	57.38	5.89	10.05
F2	3.98	2075	19.22	61.14	4.35	8.32
GBFS	15.49	2880	6.68	39.88	0.97	39.34

**Table 2:** Mortar mix design.

Mix Design (and Specimen Notation)	Supplementary Cementitious Material Type	Fly Ash [g]	GBFS [g]	Sand [g]	Sodium Silicate Solution [g]	Water [g]	Hemp Fibres [g]
FA1_0	F1	450	0	1350	306	70	-
FA1_0.5	F1	450	0	1350	306	70 + 15 *	6.25
FA1_1.0	F1	450	0	1350	306	70 + 30 *	12.50
FA2S_0	F2 + GBFS	225	225	1350	306	50	-
FA2S_0.5	F2 + GBFS	225	225	1350	306	50 + 15 *	6.25
FA2S_1.0	F2 + GBFS	225	225	1350	306	50 + 30 *	12.50

* Additional water added for fibre absorption.
